# Risk factors and prognosis of sentinel lymph node metastasis in breast-conserving breast cancer: A retrospective study based on the SEER database

**DOI:** 10.1097/MD.0000000000037263

**Published:** 2024-03-01

**Authors:** Ruihao Liu, Jian Chen, Wei Cao, Ting Li, Yulong Liao, Yingliang Li

**Affiliations:** a Emergency Department, The First Affiliated Hospital of Nanchang University Ganjiang New Area Hospital, Nanchang, China; b General Surgery Department, First Affiliated Hospital of Nanchang University, Nanchang, China; c Gynecology and Obstetrics, First Affiliated Hospital of Nanchang University, Nanchang, China.

**Keywords:** breast cancer, factor prognosis, prognostic, risk model

## Abstract

At present, the risk factors and prognosis of sentinel lymph node metastasis (SLNM) are analyzed based on the study of axillary lymph node metastasis, but whether there is a difference between the two is unclear. Therefore, an accurate and appropriate predictive model needs to be proposed to evaluate patients undergoing sentinel lymph node biopsy (SLNB) for breast cancer. We selected 16983 women with breast cancer from the Surveillance Epidemiology and End Results (SEER) database. They were randomly assigned to two cohorts, one for development (n = 11891) and one for validation (n = 5092). multi-factor logistics regression was used to distinguish risk factors affecting SLNM. The potential prognostic factors were identified using the COX regression analysis. The hazard ratio (HR) and 95% confidence interval (95%CI) were calculated for all results. Multiple Cox models are included in the nomogram, with a critical *P* value of .05. In order to evaluate the model’s performance, Concordance index and receiver operating characteristic curves were used. Six independent risk factors affecting SLNM were screened out from the Logistic regression, including tumor location, number of regional lymph nodes (2-5), ER positive, PR positive, tumor size (T2-3), and histological grade (Grade II-III) are independent risk factors for SLNM in patients (*P* < .05). Eight prognostic factors were screened out in the multivariate COX regression analysis (*P* < .05): Age: Age 60 to 79 years, Age ≥ 80 years; Race; Histological grading: Grade II, Grade III; No radiotherapy; Tumor size: T2, T3; ER positive:, sentinel lymph node positive, married. Histological grade, tumor location, T stage, ER status, PR status and the number of SLNB are significantly correlated with axillary SLNM. Age, ethnicity, histological grade, radiotherapy, tumor size, ER status, SLN status, and marital status were independent risk factors for Breast cancer specific survival (BCSS). Moreover, the survival rate of patients with 3 positive SLNs was not significantly different from that with one or two positive SLNs, We concluded that patients with stage N1 breast cancer were exempt from axillary lymph node dissection, which is worthy of further study.

## 1. Introduction

Sentinel lymph node (SLN) was found during dorsal phallic lymphangiography by Cabanas in 1977,^[1]^and was the first lymph node stop for primary tumor metastasis. When SLN did not have metastasis, there was a very low probability of non-sentinel lymph node metastasis (SLNM).^[2]^ sentinel lymph node biopsy (SLNB) provides prognostic information and guides adjuvant treatment. NSABP-B32 study confirmed that negative axillary sentinel lymph node biopsy exempted axillary lymph node dissection.^[3]^ Multiple prospective clinical studies^[4–6]^ (such as ACOSOG Z0011, AMAROS, IBCSG 23-01, etc.) have shown that For breast cancer patients with sentinel lymph node metastasis (1–2 SLNM, early breast cancer with cN0 SLN positive, sentinel lymph node micro-metastases), whether or not ALND has no significant effect on postoperative recurrence and survival. Now SLNB is recommended for clinically negative axillary patients after neoadjuvant chemotherapy.^[7]^ At present, breast-conserving surgery and SLNB have been widely recognized as radical surgical methods for patients.

With improvement of the lymph node technique and research, SLNB has been widely used in the diagnosis and treatment of cancers, especially for breast cancer, Axillary lymph node dissection (ALND) has gradually been replaced by this procedure. SLNB does not affect the diagnostic accuracy and prognostic information, and has become the standard surgical procedure for clinical lymph node negative (cN0) breast cancer patients. A number of trials have confirmed that the incidence of upper limb edema, numbness, pain, paresthesia, shoulder joint mobility disorder and other complications in SLNB patients is significantly lower than that of ALND, which improves the quality of life of patients.^[8,9]^ In the 8th edition of the American Joint Committee on Cancer (AJCC)^[10]^ breast cancer staging, the number of SLNs was less than 6, and SLN footnote “sn” could not be used if there were more than 6. But the minimum number of SLNs is not specified. So it’s theoretically possible to take one. Studies have shown that the false negative rate of sentinel lymph nodes is less than 10%.^[11]^ But surgeons tend to take more sentinel nodes during surgery to reduce the false-negative rate. There is evidence that removing fewer nodes will increase the false negative rate.^[12]^ It has been reported that the average number of SLN taken during surgery is 2.35,^[13]^ and it is believed that the number of SLN should be greater than 1. Therefore, There is an urgent need to establish an accurate model for evaluating the factors and prognosis of SLNM. Based on the Surveillance Epidemiology and End Results (SEER) database, this study collected all the information of patients with breast conserving breast cancer from 2014 to 2015, and studied the clinicopathological features, prognosis and risk factors of SLNM through the big data level. To evaluate the specific survival rate, we are working on establishing a prognostic nomogram based on important factors.

## 2. Patients and method

### 2.1. Patient data and sources

The patients were drawn from SEER database and included the all available information. In terms of demographics, these registries represent the United States’ general population.^[14]^ The inclusion criteria were as follows: diagnosed in 2014 to 2015; Confirmed surgical methods: breast conserving surgery + SLNB (code 20, 22, 23, 24), and the number of sentinel lymph nodes was 1 to 5; Clinical stage: T1-3. The chemotherapy we studied was adjuvant setting. Exclusion criteria: M1 and Mx staging; and Incomplete information.

### 2.2. Data characteristics and endpoints

The variables included: Patients’ demographics, treatment course, and tumor specific information. The primary end points were: Risk factors for SLNM. BCSS.

We convert age were transformed into categorical variables: 20 to39 years, 40 to 59 years, 60 to 79 years, ≥80years. And defined marital status as married, separated, divorced or widowed (SDW), and single. The remaining variables remain the same According to the seventh edition AJCC staging, this study documented accurate information about the TMN system.

### 2.3. Statistical analysis

We used analysis software to randomize patients into a 7 to 3 ratio between the development cohort and the validation cohort. The descriptive analysis used *t* test and Chi-square test to explore the baseline characteristics of patients in both groups. In the development cohort, multi-factor logistics regression was used to identify risk factors affecting SLNM. The potential prognostic factors were identified using the univariate COX regression analysis. The multivariate COX proportional risk regression model was used when the *P* value < .05. The hazard ratio (HR) and 95% confidence interval (95% CI) were calculated for all results. Multiple Cox models are included in the nomogram, with a critical *P* value of .05. The nomogram was created to visually predict survival probabilities in the developmental cohort. In order to evaluate the model’s performance, the Harrell’s concordance index (C-index) and receiver operating characteristic curves were used. The higher the value (close to 1), the more accurate the prediction of prognosis. Statistical analysis using R Version 4.2.2 (https://cran.r-project.org/bin/windows/base/).

## 3. Results

### 3.1. Population characteristics

Our study included 16,983 patients with breast cancer, 11,891 patients (70%) were assigned to the developmental cohort, and 5092 patients (30%) were assigned to the validation cohort. Table 1 shows the demographic and clinicopathological characteristics of the patients. The 2 groups were no statistical difference in terms of variables. For the general, developmental, and validation cohorts, the median follow-up time was 57 months. The baseline demographic and clinicopathological characteristics of positive and negative sentinel nodes in the developmental cohort are shown in Table 2. Among them, age, tumor site, histological grade, tumor size, estrogen receptor (ER) status, progesterone receptor (PR) status, the number of SLNs, and whether or not chemotherapy had statistical significance (*P* < .05).

**Table 1:** Baseline demographical and clinicopathological characteristics of patients.

Characteristics	Total cohort, N (%)	Development cohort, N (%)	Validation cohort, N (%)	*P* value
Number of patients	N = 16983	N = 11891 (70)	N = 5092 (30)	
Age				
20–39 yr	358 (2.11)	257 (2.16)	101 (1.98)	.887
40–59 yr	6179 (36.4)	4326 (36.4)	1853 (36.4)	
60–79 yr	9332 (54.9)	6533 (54.9)	2799 (55.0)	
≥80 yr	1114 (6.56)	775 (6.52)	339 (6.66)	
Race				
White	13920 (82.0)	9731 (81.8)	4189 (82.3)	.71
Black	1530 (9.01)	1087 (9.14)	443 (8.70)	
AI	112 (0.66)	75 (0.63)	37 (0.73)	
API	1421 (8.37)	998 (8.39)	423 (8.31)	
Site				
Nipple	66 (0.39)	41 (0.34)	25 (0.49)	.261
Upper-inner quadrant	3816 (22.5)	2687 (22.6)	1129 (22.2)	
Lower-inner quadrant	1559 (9.18)	1117 (9.39)	442 (8.68)	
Upper-outer quadrant	9497 (55.9)	6634 (55.8)	2863 (56.2)	
Lower-outer quadrant	2045 (12.0)	1412 (11.9)	633 (12.4)	
Histology				
Others	3729 (22.0)	2574 (21.6)	1155 (22.7)	.135
No special type	13254 (78.0)	9317 (78.4)	3937 (77.3)	
Grade				
Grade I	5243 (30.9)	3654 (30.7)	1589 (31.2)	.861
Grade II	7614 (44.8)	5346 (45.0)	2268 (44.5)	
Grade III	4126 (24.3)	2891 (24.3)	1235 (24.3)	
Laterality				
Left	8629 (50.8)	6028 (50.7)	2601 (51.1)	.645
Right	8354 (49.2)	5863 (49.3)	2491 (48.9)	
T				
T1	12920 (76.1)	9057 (76.2)	3863 (75.9)	.292
T2	3845 (22.6)	2672 (22.5)	1173 (23.0)	
T3	218 (1.28)	162 (1.36)	56 (1.10)	
Radiation				
Yes	14222 (83.7)	9966 (83.8)	4256 (83.6)	.711
No	2761 (16.3)	1925 (16.2)	836 (16.4)	
Chemotherapy				
Yes	5202 (30.6)	3655 (30.7)	1547 (30.4)	.644
No	11781 (69.4)	8236 (69.3)	3545 (69.6)	
ER				
Negative	14943 (88.0)	10452 (87.9)	4491 (88.2)	.583
Positive	2040 (12.0)	1439 (12.1)	601 (11.8)	
PR				
Negative	13400 (78.9)	9371 (78.8)	4029 (79.1)	.643
Positive	3583 (21.1)	2520 (21.2)	1063 (20.9)	
HER2				
Negative	1894 (11.2)	1344 (11.3)	550 (10.8)	.342
Positive	15089 (88.8)	10547 (88.7)	4542 (89.2)	
Regional nodes examined				
1	5254 (30.9)	3668 (30.8)	1586 (31.1)	.938
2	4977 (29.3)	3487 (29.3)	1490 (29.3)	
3	3525 (20.8)	2485 (20.9)	1040 (20.4)	
4	1998 (11.8)	1388 (11.7)	610 (12.0)	
5	1229 (7.24)	863 (7.26)	366 (7.19)	
Positive lymph nodes				
No	13631 (80.3)	9553 (80.3)	4078 (80.1)	.706
Yes	3352 (19.7)	2338 (19.7)	1014 (19.9)	
Marital status				
Single	2324 (13.7)	1626 (13.7)	698 (13.7)	.025
Married	10180 (59.9)	7198 (60.5)	2982 (58.6)	
SDW	4479 (26.4)	3067 (25.8)	1412 (27.7)	

AI = American Indian/Alaska Native, API = Asian or Pacific Islander, SDW = separated, divorced or widowed.

**Table 2:** Clinicopathological parameters of included patients and association with SLN status.

	Negative (N = 9553)	Positive (N = 2338)	*P* value
Age			
20–39 yr	190 (2.0%)	67 (2.9%)	<.001
40–59 yr	3398 (35.6%)	928 (39.7%)	
60–79 yr	5341 (55.9%)	1192 (51.0%)	
≥80 years	624 (6.5%)	151 (6.5%)	
Race			
W	7831 (82.0%)	1900 (81.3%)	.0517
B	842 (8.8%)	245 (10.5%)	
AI	61 (0.6%)	14 (0.6%)	
API	819 (8.6%)	179 (7.7%)	
Site			
Nipple	26 (0.3%)	15 (0.6%)	<.001
Upper-inner quadrant	2314 (24.2%)	373 (16.0%)	
Lower-inner quadrant	920 (9.6%)	197 (8.4%)	
Upper-outer quadrant	5188 (54.3%)	1446 (61.8%)	
Lower-outer quadrant	1105 (11.6%)	307 (13.1%)	
Histologic			
Others	2082 (21.8%)	492 (21.0%)	.446
No special type	7471 (78.2%)	1846 (79.0%)	
Grade			
Grade I	3143 (32.9%)	511 (21.9%)	<.001
Grade II	4171 (43.7%)	1175 (50.3%)	
Grade III	2239 (23.4%)	652 (27.9%)	
Laterality			
Left	4859 (50.9%)	1169 (50.0%)	.468
Right	4694 (49.1%)	1169 (50.0%)	
T			
T1	7675 (80.3%)	1382 (59.1%)	<.001
T2	1779 (18.6%)	893 (38.2%)	
T3	99 (1.0%)	63 (2.7%)	
Radiation			
Yes	7999 (83.7%)	1967 (84.1%)	.661
No	1554 (16.3%)	371 (15.9%)	
Chemotherapy			
Yes	2423 (25.4%)	1232 (52.7%)	<.001
No	7130 (74.6%)	1106 (47.3%)	
ER			
Negative	8350 (87.4%)	2102 (89.9%)	.001
Positive	1203 (12.6%)	236 (10.1%)	
PR			
Negative	7445 (77.9%)	1926 (82.4%)	<.001
Positive	2108 (22.1%)	412 (17.6%)	
HER2			
Negative	1068 (11.2%)	276 (11.8%)	.413
Positive	8485 (88.8%)	2062 (88.2%)	
Examined			
1	3117 (32.6%)	551 (23.6%)	<.001
2	2837 (29.7%)	650 (27.8%)	
3	1942 (20.3%)	543 (23.2%)	
4	1044 (10.9%)	344 (14.7%)	
5	613 (6.4%)	250 (10.7%)	
Marital			
Single	1314 (13.8%)	312 (13.3%)	.154
Married	5743 (60.1%)	1455 (62.2%)	
SDW	2496 (26.1%)	571 (24.4%)	

AI = American Indian/Alaska Native, API = Asian or Pacific Islander, SLN = sentinel lymph node.

### 3.2. Analysis of factors influencing SLNM in a developmental cohort

In Table 2, there were 7 statistically significant factors: age, tumor site, histological grade, tumor size, ER status, PR status, and the number of SLNs. It has been previously reported that the expression of human epidermal growth factor receptor-2^[15]^ may also affect axillary metastasis in patients. These 8 factors were used as independent variables and SLNM was used as the dependent variable in a multivariate binary logistic regression analysis. Finally, 6 independent risk factors affecting SLNM were screened out from the multivariate Logistic regression analysis, including tumor location (upper inner quadrant, lower inner quadrant, upper outer quadrant, Outer and lower quadrant), number of regional lymph nodes examined (2-5), ER positive, PR positive, tumor size (T2-3), and histological grade (Grade II-III) are independent risk factors for SLNM in patients. (Table 3).

**Table 3:** Multivariate analysis of SLNM.

	Pr(> z )	OR	2.50%	97.50%
Age 40–59 yr	0.599	0.922	0.686	1.25
Age 60–79 yr	0.239	0.835	0.621	1.13
Age ≥ 80 yr	0.344	0.846	0.599	1.2
Site Upper-inner quadrant	<0.01	0.281	0.145	0.563
Site Lower-inner quadrant	<0.01	0.4	0.204	0.808
Site Upper-outer quadrant	0.044	0.504	0.262	1
Site Lower-outer quadrant	0.047	0.504	0.259	1.01
Node examined 2	<0.01	1.27	1.11	1.44
Node examined 3	<0.01	1.52	1.33	1.74
Node examined 4	<0.01	1.77	1.51	2.07
Node examined 5	<0.01	2.22	1.86	2.66
ER Positive	<0.01	0.704	0.571	0.866
PR Positive	<0.01	0.668	0.568	0.783
HER2 Positive	0.071	1.15	0.989	1.33
T2	<0.01	2.68	2.41	2.9
T3	<0.01	3.49	2.5	4.85
Grade II	<0.01	1.56	1.38	1.75
Grade III	<0.01	1.65	1.42	1.92

SLNM = sentinel lymph node metastasis.

### 3.3. Prognostic factors of patients in development cohort

In multivariate COX analysis, 8 prognostic factors were selected, including age, race, histological grade, radiotherapy, tumor size, ER status, SLN positive status and marital status. The 8 independent factors were identified (*P* ≤ .05): Age: Age 60 to 79 years, Age ≥ 80 years; Race: Black, American Indian/Alaska Native (AI), Asian or Pacific Islander (API); Histological grading: Grade II, Grade III; No radiotherapy; Tumor size: T2, T3; ER positive: sentinel lymph node positive, married (Table 4).

**Table 4:** Univariate and multivariate regression analyses for BCSS.

Characteristics	Univariate analysis HR (95% CI)	*P* value	Multivariate analysis HR (95% CI)	*P* value
Age				
Age 20–39 yr	Ref.		Ref.	
Age 40–59 yr	0.888 (0.482–1.634)	.702	1.167 (0.632–2.155)	.595
Age 60–79 yr	1.806 (0.994–3.282)	.052	2.489 (1.357–4.564)	.002 *
Age ≥ 80 yr	6.357 (3.463–11.670)	<.001*	6.536 (3.487–12.250)	<.001*
Race				
White	Ref.		Ref.	
Black	1.647 (1.363–1.989)	<.001*	1.454 (1.194–1.770)	<.001*
AI	1.776 (0.921–3.428)	.087	2.061 (1.065–3.986)	.035*
API	0.650 (0.483–0.872)	.004*	0.738 (0.548–0.993)	.041*
Site				
Nipple	Ref.			
Upper-inner quadrant	0.531 (0.219–1.292)	.163		
Lower-inner quadrant	0.639 (0.260–1.573)	.33		
Upper-outer quadrant	0.611 (0.253–1.475)	.273		
Lower-outer quadrant	0.560 (0.228–1.376)	.206		
Histologic type				
Others	Ref.			
No special type	1.148 (0.973–1.355)	.103		
Grade				
Grade I	Ref.		Ref.	
Grade II	1.311 (1.100–1.562)	.002*	1.209 (1.011–1.445)	.0344 *
Grade III	2.329 (1.951–2.782)	<.001*	2.031 (1.647–2.504)	<.001*
Laterality				
Left	Ref.			
Right	0.975 (0.855 –1.112)	.701		
Radiation				
Yes	Ref.		Ref.	
No	2.362 (2.044–2.728)	<.001*	1.812 (1.560–2.104)	<.001*
Chemotherapy				
Yes	Ref.			
No	0.947 (0.822–1.090)	.448		
T				
T1	Ref.		Ref.	
T2	1.833 (1.593–2.109)	<.001*	1.498 (1.287–1.744)	<.001*
T3	2.519 (1.674–3.789)	<.001*	2.277 (1.492–3.475)	<.001*
ER				
Negative	Ref.		Ref.	
Positive	1.917 (1.627–2.259)	<.001*	1.387 (1.085–1.773)	.009*
PR				
Negative	Ref.		Ref.	
Positive	1.67 (1.447–1.927)	<.001*	1.170 (0.953–1.436)	.162
HER2				
Negative	Ref.			
Positive	1.01 (0.819–1.244)	.927		
Node Examined				
	Ref.			
2	0.994 (0.840–1.177)	.947		
3	0.944 (0.782–1.139)	.549		
4	1.075 (0.863–1.339)	.518		
5	0.864 (0.650–1.149)	.316		
Node positive				
Negative	Ref.		Ref.	
Positive	1.502 (1.294–1.743)	<.001*	1.4981 (1.281–1.752)	<.001*
Marital				
Single	Ref.		Ref.	
Married	0.669 (0.549–0.816)	<.001*	0.719 (0.586–0.881)	.001*
SDW	1.531 (1.254–1.870)	<.001*	1.149 (0.932–1.415)	.193

AI = American Indian/Alaska Native, API = Asian or Pacific Islander, SDW = separated, divorced or widowed.

### 3.4. Prognostic nomogram for CSS

**Figure 1.**
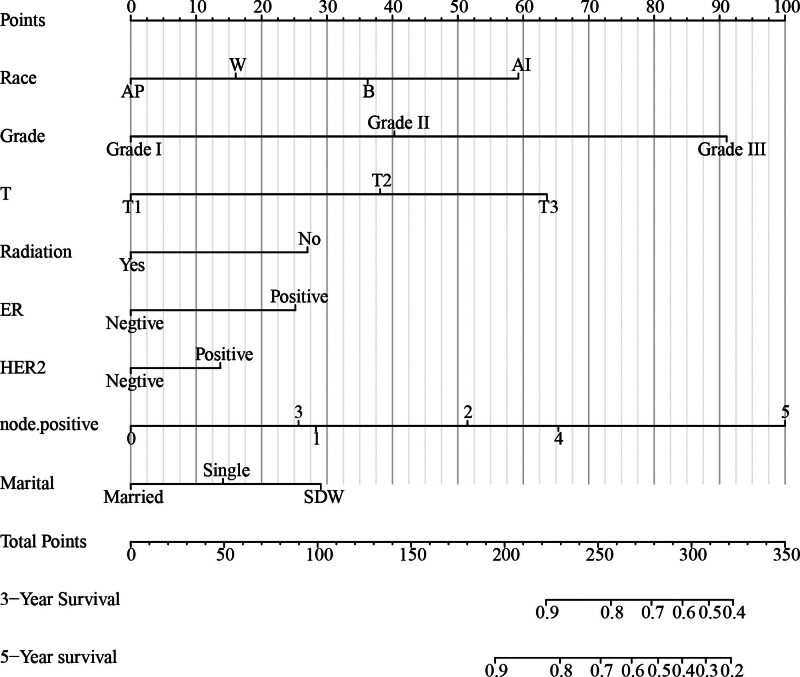
Nomogram predicting 3-year and 5-year cancer-specific survival probability for BCSS.

According to the COX regression analysis, nomogram predicted 3-year and 5-year CSS for breast cancer patients (Fig. 1). As a result of the contribution to the nomogram, a corresponding score is assigned to all variables in the nomogram, ranging from 0 to 100. Patients can get an overall score by adding the scores for each subgroup.

### 3.5. Feasibility of the nomogram

**Figure 2.**
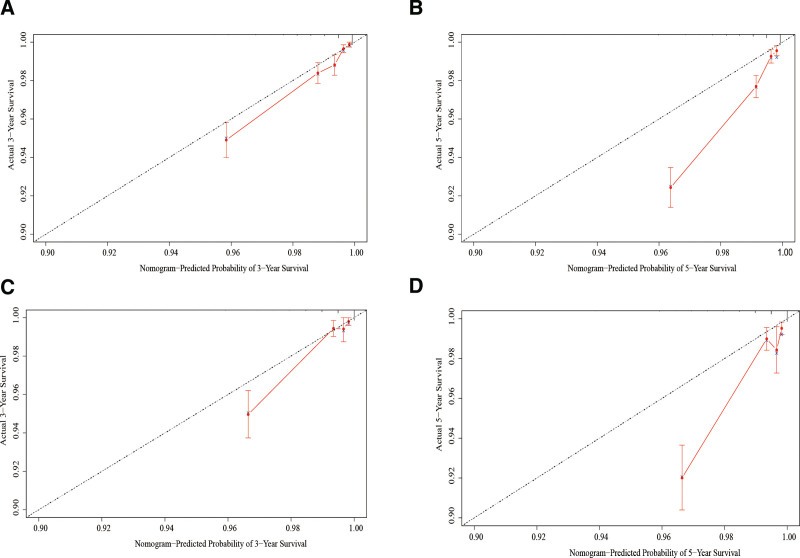
ROC curves of the nomogram predicting 3-year (A) and 5-year (B) BCSS in the development cohort; 3-year (C) and 5-year (D) BCSS in the validation cohort. ROC = receiver operating characteristic.

**Figure 3.**
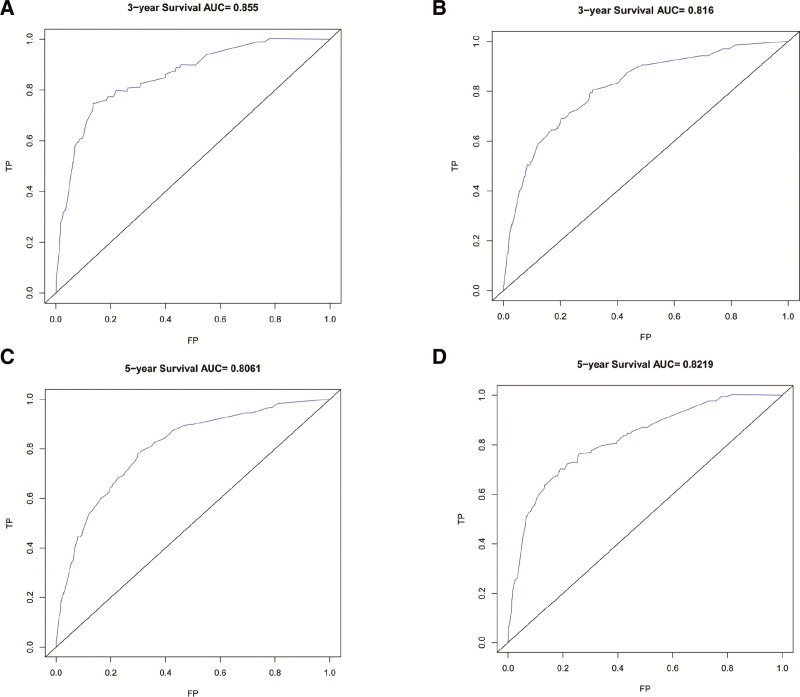
C-index of the nomogram predicting 3-year (A) and 5-year (B) BCSS in the development cohort; 3-year (C) and 5-year (D) BCSS in the validation cohort.

The C index was 0.807 (0.782–0.832) In the development cohort. At the same time, receiver operating characteristic curve was used to evaluate the discriminant ability of the model. AUC values were significantly higher for both 3-year (0.816) and 5-year (0.806) forecasts (Fig. 2A and B). According to the calibrated graphs for both the 3-year and 5-year development cohorts, actual observations and predictions are in good agreement (Fig. 3A and B), As a result, we can conclude that our model has relatively good performance. In addition, A validation queue was used to evaluate the nomogram’s applicability. The C index is 0.826 (0.790–0.863), AUC values were significantly higher for both 3-year (0.816) and 5-year (0.806) forecasts (Fig. 2C and D). The calibration curves of the verification queue predict the results well and are in good agreement with the actual results. Results from internal validation indicated that the diagram was of satisfactory applicability to patients with SLNB (Fig. 3C and D).

### 3.6. Survival curve for nomogram

**Figure 4.**
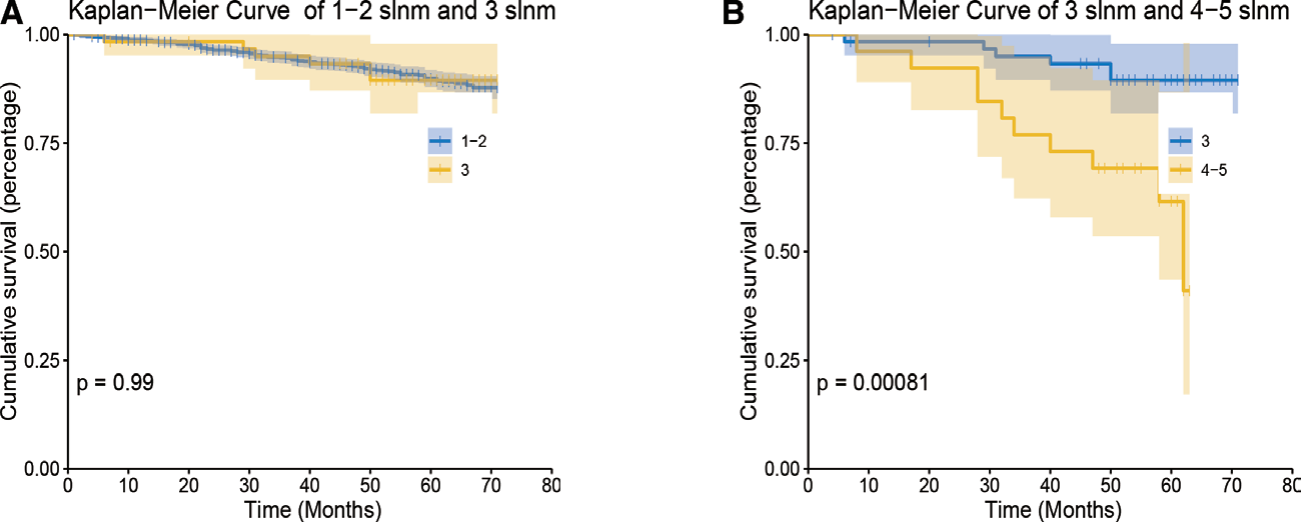
BCSS curves of different positive SLNs were calculated using the Kaplan–Meier cure.

We followed the patients for a median of 57 months (0–71 months). We observed that 889 patients died, including 304 breast cancer specific deaths. The 3-year BCSS rates were 98.5%, and the 5-year BCSS rates were 97.6%. In order to observe the survival of different numbers of SLNM, BCSS curves of different positive SLNs were calculated using the Kaplan–Meier curve (Fig. 4). We observed significant differences between different amounts of SLNM (*P* < .001)

## 4. Discussion

Based on the large clinical studies represented by SENTINA and ACOSOG Z0011, breast conserving surgery combined with SLNB has become the mainstream operation at present, which has been widely recognized by clinicians. The latest guidelines recommend that patients with one or two SLNM who have received breast-conserving therapy and postoperative radiation therapy should avoided ALND.^[16]^ At present, the risk factors and prognosis of SLNM are analyzed based on the study of axillary lymph node metastasis, But it is unclear whether there is difference. Therefore, an accurate and appropriate predictive model needs to be proposed for the evaluation of breast cancer patients performing SLNB.

Comprehensive analysis of all the available factors in the SEER database was conducted. The rate of SLNM was 19.7% in the study. Among the samples with SLNM, the proportion of biopsy was 23.6% for one SLN, 27.8% for 2 SLNs, 23.2% for SLNs, 14.7% for SLNs, and 10.7% for 5 SLNs. The results showed that histological grade, tumor location, T stage, ER status, PR status, and the number of SLNs were significantly correlated with SLNM. Except for the number of SLN, the results are similar to those of domestic and foreign studies on axillary lymph node metastasis.^[17,18]^ Although the false negative rate of one SLN biopsy during SLNB operation was less than 10%,^[11,19]^ in this study, The number of 2 to 5 SLNs is an independent risk factor for positive SLN by logistic regression, and according to the NCCN(National Comprehensive Cancer Network) guidelines, breast conserving breast cancer patients with one to two SLNM can be exempted from ALND surgery. Therefore, when performing SLNB surgery, we should biopsy two or more SLNs to accurately predict whether patients have non-SLNM and whether ALND surgery can be exempted.

The latest prospective trial^[7]^showed that after neoadjuvant chemotherapy, The false negative rate of SLN was < 10% when there were 3 or more SLNs. This also has reference value for the results of this study. The combined tracer technique is the gold standard for SLNB and can achieve a detection rate of more than 95% and a false negative rate of less than 10%.^[20]^ Therefore, the use of dual tracer technology can improve the accuracy of our experiment.

We constructed nomogram using variables from the multifactor COX model and used it to predict BCSS. With this approach, an accurate tool was produced that accurately included only variables associated with survival. the survival nomogram was successfully constructed with relatively good predictability. another advantage of the nomogram compared with the multiple regression is that it provides the probability of individual survival outcomes at a specific point in time, rather than the relative risk concept. Meanwhile, compared with the traditional COX regression model,^[21,22]^ nomogram’s accuracy can also be evaluated using Harrell’s C-index.

In the multivariate COX model, age, ethnicity, histological grade, radiotherapy, tumor size, ER status, SLNM status and marital status were independent prognostic factors for BCSS. Although the ACOSOG Z0011 trial confirmed that there was no significant difference in 10-year survival rate between SLNB and ALND for patients with 1 to 2 SLNM, N1 included 1 to 3 SLNM patients according to the eighth edition of AJCC breast cancer Staging Guidelines.^[10]^ Therefore, We also studied the effect of more than two SLNs positive on survival and prognosis of patients. Interestingly, the risk score of 3 SLNs was slightly lower than that of one SLN in nomogram, and the Kaplan–Meier method obtained different BCSS curves with positive SLNs. Kaplan–Meier method was used to compare the survival curves of 1 to 2 SLNM patients with 3 SLNM patients, 3 SLNM patients with 4 to 5 SLNM patients, respectively. During the follow-up period, there was no significant difference in the survival rate between patients with 3 SLNM and those with 1 to 2 SLNM (*P* > .05) (Fig. 4A), while there was a significant difference between patients with 3 SLNMs and those with 4 to 5 SLNMs (*P* < .05) (Fig. 4B). According to the diagram, The survival rate of patients who had 3 SLNM were not significantly different from that with one or two SLNM. So are N1 breast cancer patients exempt from ALND surgery?C. Bonneau et all found that patients with T1T2 invasive breast cancer with 3 lymph node metastases did not benefit from ALND after SLNB, and ALND was limited to staging.^[23]^ Yun Fu et al^[24]^ suggested that radiotherapy after SLNB could replace ALND in patients with N1 breast cancer. The results showed that patients with stage N1 could be exempted from ALND after SLNB. The difference between the results of other studies and the ACOSOG Z0011 trial may be due to the fact that patients with 1-2 SLNM were included in the ACOSOG Z0011 trial, and patients with 3 SLNM and only SLNB were not included in the study. However, ACOSOG Z0011 is a prospective study. Other studies are retrospective and have certain limitations. At the same time, the AMAROS trial confirmed that radiotherapy can achieve the same control effect as ALND in SLNM cT1-2 breast cancer patients.^[25]^ However, we generally believe that the more lymph node metastases, the worse the prognosis.^[26]^ Therefore, we should conduct prospective studies for further verification.

This study uses SEER database to provide a large sample for analysis, but it still has drawbacks. First, the results are inevitably affected by selection bias. For example, the SEER database collects a large number of patients information from multiple regions and hospitals. Doctors have certain differences in the treatment methods of patients, such as the dosage of therapeutic drugs and radiotherapy may be different. Lastly, even though internal validation was performed, As a result of using the same database for both development and validation, the results were not perfect. For external validation, a large prospective clinical trial is required.

## 5. Conclusion

This SEER database-based study revealed demographic, clinicopathological and therapeutic characteristics that were significantly associated with specific survival of breast cancer patients undergoing sentinel node biopsy. We constructed and validated prognostic nomogram to predict individualized probabilities of 3-year and 5-year specific survival in breast cancer patients. Nomogram facilitates patient consultation, follow-up planning and treatment selection. We also concluded that patients with stage N1 breast cancer were exempt from axillary lymph node dissection. However, Whether the clinical operation can be downgraded needs to be validated prospectively.

## Author contribution

**Data curation:** Ruihao Liu, Yulong Liao.

**Formal analysis:** Jian Chen, Ting Li, Yulong Liao.

**Investigation:** Ting Li.

**Methodology:** Wei Cao, Yingliang Li.

**Software:** Ruihao Liu, Wei Cao.

**Supervision:** Yingliang Li.

**Visualization:** Jian Chen.

**Writing – original draft:** Ruihao Liu.

**Writing – review & editing:** Yingliang Li.
